# Unusual behaviour of the spin-phonon coupling in the quasi-one-dimensional antiferromagnet RbCoCl_3_

**DOI:** 10.1038/s41598-022-18073-3

**Published:** 2022-08-18

**Authors:** M. G. Cottam, D. J. Lockwood

**Affiliations:** 1 Department of Physics and Astronomy, University of Western Ontario, London, ON N6A 3K7 Canada; 2 Metrology Research Centre, National Research Council, Ottawa, ON K1A 0R6 Canada

**Keywords:** Materials science, Optics and photonics, Physics

## Abstract

We present an experimental and theoretical study for the lattice vibrational (phonon) modes in the quasi-one-dimensional (or chain-like) antiferromagnet RbCoCl_3_ at low temperatures both above and below the two different magnetic phase transitions. Clear evidence is found for the role of spin-phonon interactions in providing a temperature-dependent contribution for the frequencies of the E_1g_ and E_2g_ symmetry phonons that occur with frequencies comparable to those of the spin wave excitations (magnons) in this compound. The behaviour in RbCoCl_3_, as studied here by Raman scattering experiments, is quite different from that typically observed in rutile-structure antiferromagnets where the spin-phonon coupling has been well characterized. The theory is modified to take account of the strong Ising-like component in the spin Hamiltonian. This enables the spin-phonon coupling parameters to be deduced, with the analysis also revealing the onset of an extra frequency shift for the phonons below the transition temperature *T*_*N*1_ = 28 K associated with magnetic ordering along the Co chains.

## Introduction

Spin-phonon coupling in magnetic solids is ubiquitous^1^ and is important for many of the physical properties of such solids. The signature of the sometimes strong, but usually subtle, interaction between lattice vibrational modes (phonons) and spin waves (magnons) in the magnetically ordered state can take various forms that are often best studied at near-zero wave vector, which makes them ideal for optical investigations using inelastic light scattering (Raman or Brillouin spectroscopy)^1^ or infrared absorption. A thorough knowledge of such spin-phonon coupling is very important for understanding the full physical properties in the dynamics of magnetic materials and can be the determining factor for the applications of certain materials in practical applications^2–4^.

**Figure 1:**
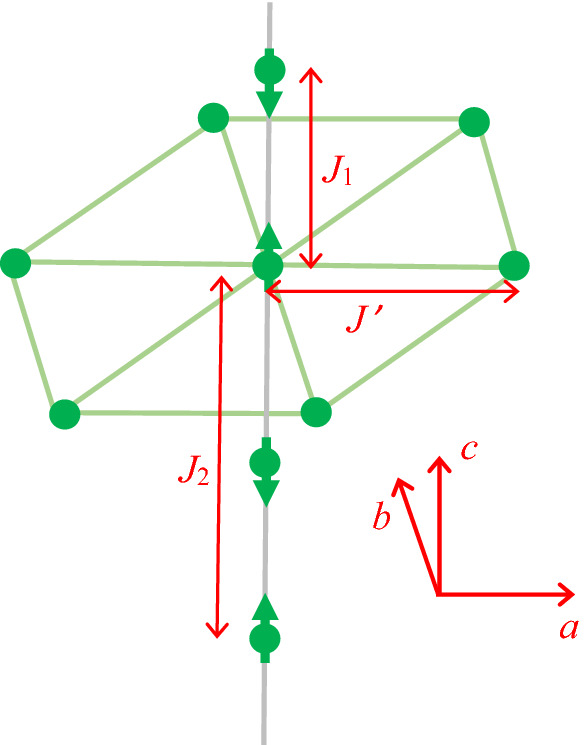
Schematic of crystal structure of RbCoCl_3_, showing the Co ions only (filled green circles) ordered antiferromagnetically along chains (in the crystallographic *c* direction) with nearest-neighbour exchange *J*_1_ and next nearest-neighbour exchange *J*_2_. Each Co ion is surrounded by a hexagon of six Co ions with interchain exchange interaction *J’* in the *a-b* plane.

There has been sustained interest in recent years regarding the novel properties of hexagonal perovskite compounds of the form ABX_3_, where A is Cs, Rb or Tl, B is a divalent metal ion, and X is an alkali halide (typically Cl or Br). The hexagonal crystal structure is as shown in outline in Fig. 1, where the positions of the magnetic B atoms (Co in this case) have been emphasized. Such perovskites containing spin-1/2 magnetic Co ions have been found to exhibit quasi one-dimensional (1D) antiferromagnetic ordering at low temperatures as a result of dominant Ising model exchange interactions occurring along the chains of Co magnetic ions (see Fig. 1). Features of interest include spin-wave energy continua and the possibility of bound magnon states and their potential applications in spin-1/2 quantum-wire transport devices. Rubidium cobalt chloride (RbCoCl_3_) is an example of such a desirable magnetic material, because of its classic quasi 1D magnetic ordering^5,6^, and will be studied here for that reason. Most of these transition metal perovskites exhibit two magnetic phase transitions at temperatures denoted by *T*_*N*1_ and *T*_*N*2_. respectively. The higher of these transition temperatures, *T*_*N*1_, represents the temperature below which there is antiferromagnetic ordering in one dimension along the chains, whereas the lower temperature *T*_*N*2_ signifies the onset of an additional 3D inter-chain ordering due to exchange interactions between Co ions within crystalline *a-b* planes (see Fig. 1). A large amount of neutron scattering work, as well as optical measurements such as Raman scattering and far infrared spectroscopy, for these compounds have been used to determine their structural and dynamic properties. These experimental studies have included CsCoCl_3_ (see, e.g.^7–11^), RbCoCl_3_^12–15^, TlCoCl_3_^16^, and CsCoBr_3_^8–10,17–19^.

It has been shown from symmetry considerations that, given the D_6h_ hexagonal symmetry of the crystalline unit cell, the Raman active phonons at zero wave vector in RbCoCl_3_ comprise the A_1g_, E_1g_ and three E_2g_ modes^13^. Symmetry coordinates for all 30 different vibrational modes at zero wave vector in crystals with the same structure as RbCoCl_3_ have been determined^20^. Polarized Raman scattering has been used to identify the various phonons, and at ~ 11 K their frequencies are 60.6 (E_2g_ mode), ~ 115 (E_1g_), 132.1 (E_2g_), 192.9 (E_2g_), and 272.6 (A_1g_) cm^−1^^13^.

Here, we use Raman scattering to demonstrate experimentally that spin-phonon coupling is significant in RbCoCl_3_ and has unusual characteristics. We employ a theoretical model that has been adapted from earlier work to determine the coupling strengths. We find indeed that the coupling is significant and is strong enough to warrant serious consideration in developing applications of interest vis-à-vis the physical properties of 1D ordered RbCoCl_3_.

## Results

### Experiment

The three vibrational modes of E_2g_ symmetry in RbCoCl_3_ can be expected to be observed in (ZX) polarization along with the magnetic excitations^6,13^. Although the original Raman study of RbCoCl_3_ was a very general one^13^, the X(ZX)Y scattering geometry was selected for a detailed characterization of the magnetic excitations as a function of temperature. Under these experimental circumstances, only the E_1g_ mode and one of the E_2g_ modes at 132.1 cm^−1^, which appears weakly in the X(ZX)Y spectrum via polarization leak-through arising from imperfect experimental conditions, have been studied in the detail required for an analysis of their spin-phonon coupling. The sample temperatures reported here, when compared with those reported in^13^, have been corrected for a laser heating of 8 K, which was determined from a preliminary analysis of the temperature dependences of the magnon peak parameters, where the supposed temperatures of *T*_*N*1_ and *T*_*N*2_ were found to be 20 K and 4 K, respectively, for the accepted temperature values of 28 K and 12 K, respectively.

**Figure 2:**
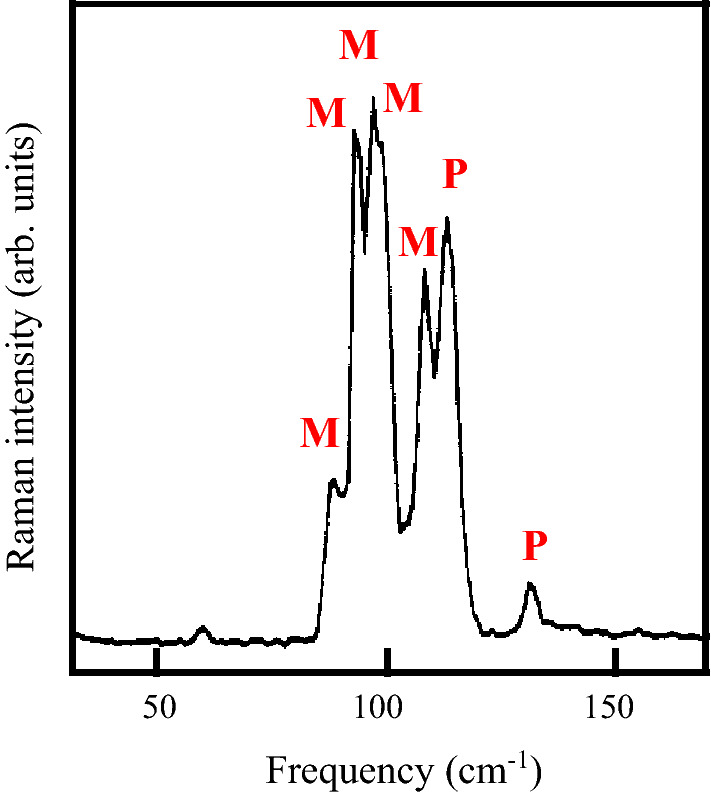
The low-frequency Raman spectrum of RbCoC1_3_ recorded with a spectral resolution of 2.2 cm^−1^ at ~ 11 K in X(ZX)Y polarization. The Raman peaks labelled M are magnetic excitations that have been evaluated elsewhere^6^, and those labelled P are the phonons of interest here.

A typical result obtained for measurements of the X(ZX)Y polarized Raman spectrum of RbCoCl_3_ at low temperature and at lower frequency is shown in Fig. 2. The phonon peaks have been curve fitted with an anharmonic oscillator model to ascertain their mode frequency, damping, and strength (following Ref.^13^). It is seen that two of the phonon modes (the E_1g_ mode and an E_2g_ mode) occur in close proximity to magnon peaks, making them promising candidates for spin-phonon studies.

### Theory

It is known that the magnetic behaviour of RbCoCl_3_ can typically be represented by a spin Hamiltonian^6,13^ where the dominant term is

1 $$2{J}_{1}\sum_{j}\left[\alpha {\mathbf{S}}_{j}\cdot {\mathbf{S}}_{j+1}+\left(1-\alpha \right){S}_{j}^{z}{S}_{j+1}^{z}\right].$$

Here *J*_1_ > 0 is the nearest-neighbour exchange interaction between spin vectors labelled as **S**_*j*_ and **S**_*j*+1_ along a chain of Co ions (see Fig. 1). There is a linear combination of Heisenberg and Ising terms, proportional to ${\mathbf{S}}_{j}\cdot {\mathbf{S}}_{j+1}$ and ${S}_{j}^{z}{S}_{j+1}^{z}$ respectively, where it has been estimated from neutron and Raman scattering data^5,6^ that *α* = 0.112. The spin Hamiltonian also contains a similar term describing the anisotropic (Heisenberg plus Ising) exchange interaction *J*_2_ to next-nearest neighbours along a chain. The role of the interchain exchange interaction *J’*, which is much weaker, is mainly in causing a discretization of the band of magnons^6^.

The basic mechanism for the spin-phonon interaction (following previous studies mainly on rutile-structure antiferromagnets^21,22^) is that, if we consider a bilinear exchange term of the general form $2J\left[\alpha {\mathbf{S}}_{1}\cdot {\mathbf{S}}_{2}+\left(1-\alpha \right){S}_{1}^{z}{S}_{2}^{z}\right]$, the exchange *J* between the two sites labelled 1 and 2 depends on the instantaneous coordinates of those sites, as well as the coordinates of other sites (for the Rb and Cl ions in this case) because of super-exchange effects. When a Taylor series expansion about the equilibrium positions is made for this modulated exchange, there will be interaction terms that are linear in the displacements (and hence in the phonon amplitudes) and depend quadratically on the spins through the combination of terms appearing above. Thus, the renormalized frequency ${\omega }_{ph}$ of any phonon can be written in first order of perturbation theory as

2 $${\omega }_{ph}={\omega }_{ph}^{0}+\lambda \langle \alpha {\mathbf{S}}_{1}\cdot {\mathbf{S}}_{2}+\left(1-\alpha \right){S}_{1}^{z}{S}_{2}^{z}\rangle,$$

where ${\omega }_{ph}^{0}$ is the phonon frequency in the absence of spin–phonon coupling and *λ* is a constant (either positive or negative, depending on the phonon mode). The angular brackets around the spin products denote a statistical average involving the pairs of neighbouring spin sites. This quantity is approximately equal to –*S*^2^ in the zero-temperature limit and its magnitude decreases monotonically with increasing *T*, becoming small (but nonzero) above *T*_*N*1_. It is convenient to write the above frequency shift as

3 $$\Delta {\omega }_{ph}\equiv {\omega }_{ph}-{\omega }_{ph}^{0}=-\lambda {S}^{2}\Phi \left(T\right),$$

where we have introduced a pair correlation function by

4 $$\Phi \left(T\right)=-\frac{1}{{S}^{2}}\langle \alpha {\mathbf{S}}_{1}\cdot {\mathbf{S}}_{2}+\left(1-\alpha \right){S}_{1}^{z}{S}_{2}^{z}\rangle >0.$$

**Figure 3:**
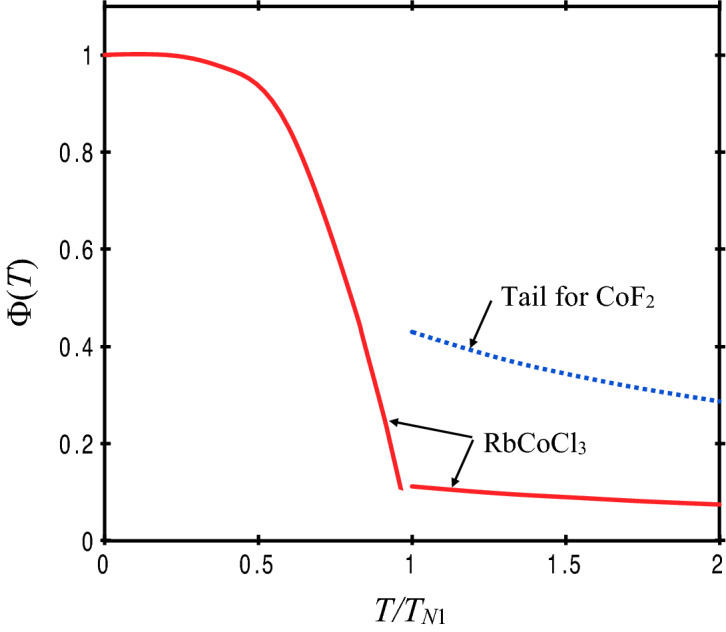
The *T*-dependence of $\Phi \left(T\right)$ estimated for RbCoC1_3_ at temperatures *T* below and above *T*_N1_. A comparison with CoF_2_ at high temperatures is also shown.

This generalizes the definition given in our earlier work^21,22^ to the case where $\alpha \ne 1$, i.e., to include an Ising component to the bilinear exchange interaction. The numerical calculation for the *T*-dependence of $\Phi \left(T\right)$ may now be carried out following the method described previously, i.e., based on a modification of the so-called constant coupling approximation introduced in^23^. The resulting estimate for RbCoCl_3_ is shown in Fig. 3 (solid lines), taking account of the exchange coupling to the intrachain exchange interactions *J*_1_ and *J*_2_. For comparison, the result for another Co compound, the rutile-structure antiferromagnet CoF_2_, is also shown for temperatures above its *T*_*N*_ value. We note that the high-temperature ‘tail’ is quite small for RbCoCl_3_ (being only ~ 10% or less of the low-temperature value for Φ). This is mainly due to the relatively large Ising component of the exchange in RbCoCl_3_ as compared to the case of CoF_2_.

## Discussion

**Figure 4:**
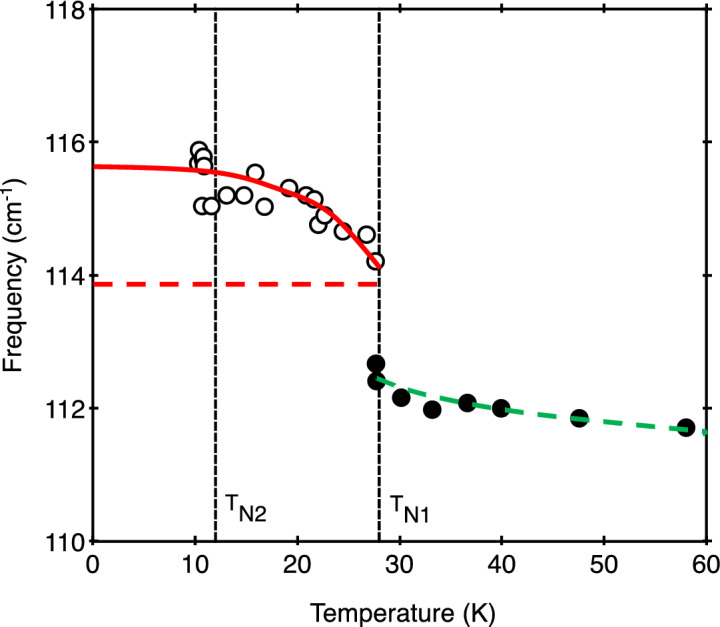
Temperature dependence of the E_1g_ mode frequency (data points) from Raman scattering measurements. The other lines are guides to the eye and are used in the theory (see the text).

**Figure 5:**
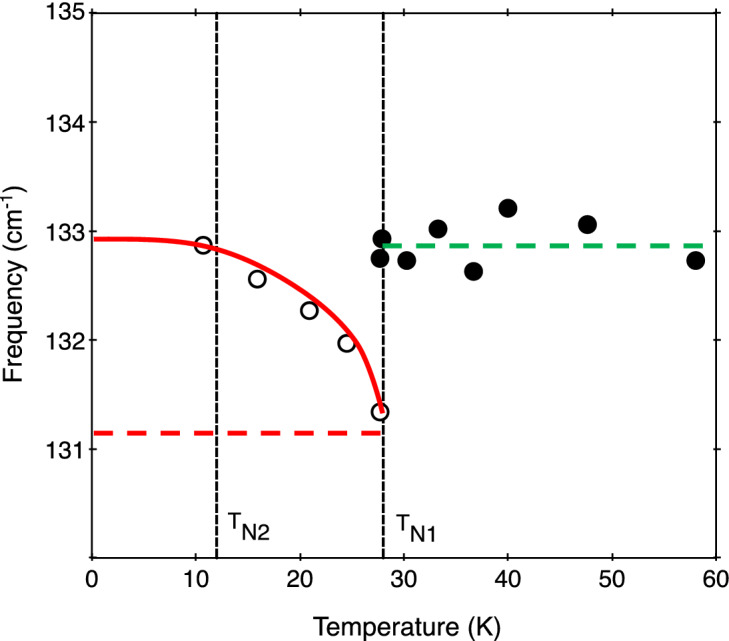
The same as in Fig. 4, but for the E_2g_ phonon mode frequency.

The main results are for the dependence of the phonon frequencies on temperature, as observed by Raman scattering and interpreted by the theory just described. Figures 4 and 5 show the frequencies plotted versus temperature for the E_1g_ mode and E_2g_ (132.1 cm^−1^) mode, respectively. In both cases there is a clear and abrupt effect occurring in the measured data in the vicinity of the temperature *T*_*N*1_, below which the 1D antiferromagnetic order sets in along the Co chains. To provide interpretation we have drawn some guide-to-the-eye lines in these figures. First, we note that the solid red line drawn through the data for *T* < *T*_*N*1_ has just the behaviour displayed in Fig. 3 for the spin–spin correlation function $\Phi \left(T\right)$. The horizontal dashed red line provides an estimate for the $\Phi =0$ base line representing the absence of spin-phonon coupling (after allowing for a high-temperature tail, as in Fig. 3). A comparison with Eq. (3) then allows us to deduce the spin-phonon coupling parameter *λ* for each phonon mode. We obtain the approximate values *λS*^2^ = − 1.8 cm^−1^ and − 1.6 cm^−1^ for the E_1g_ and E_2g_ phonons, respectively, which are of comparable magnitude to values previously found for rutile-structure antiferromagnets^21,22^. In RbCoCl_3_, however, there is an additional effect evident at *T*_*N1*_. This is evident on observing an unusual mismatch between the horizontal dashed red line below *T*_*N1*_ and the corresponding phonon frequency just above *T*_*N1*_ (as represented by the dashed green line). There is a “shift” (discontinuity) of about 1.8 cm^−1^ at *T*_*N1*_ for the E_1g_ mode, whereas for the E_2g_ mode the shift has the opposite sign and a magnitude of about 1.5 cm^−1^.

The data shown in Figs. 4 and 5 therefore reveal that, in addition to a spin-phonon interaction in RbCoCl_3_, the quasi-1D ordering at *T*_N1_ induces a structural phase transition. The abrupt shift in phonon frequency of both the E_1g_ and E_2g_ modes indicates that the transition is first-order in nature. The relative shift in frequency at *T*_N1_ when the temperature is lowered is + 1.8 cm^−1^ for the E_1g_ phonon and − 1.5 cm^−1^ for the E_2g_ phonon. Given that the normal modes for the A_1g_ and E_1g_ modes^20^ comprise Cl-ion motions perpendicular and parallel to the *c* axis, respectively, while the E_2g_ modes involve both Cl- and Rb-ion displacements within hexagonal planes, we conclude that the crystal lattice contracts a little along the c axis while expanding in the *a-b* plane when the temperature is lowered to *T*_*N*1_ and below. The lack of an observable mode splitting of the E_2g_ mode at *T*_*N*1_ and lower temperatures indicates that the crystal still has a hexagonal structure or has been lowered to that of a triclinic structure rather than a possible monoclinic structure.

**Figure 6:**
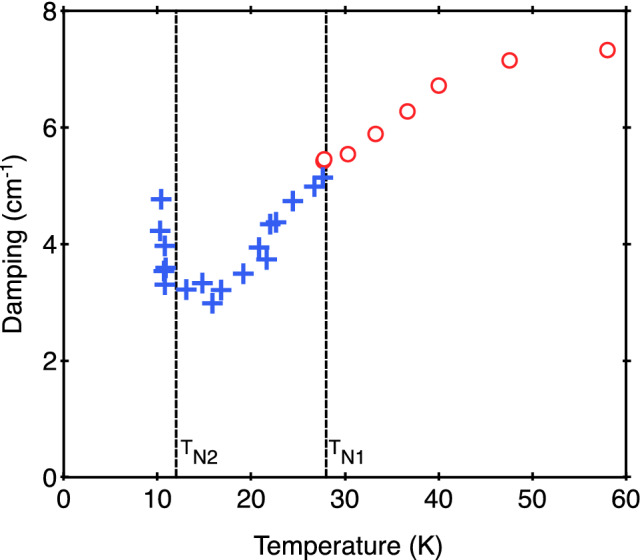
Temperature dependence of the E_1g_ mode line width from Raman scattering measurements.

**Figure 7:**
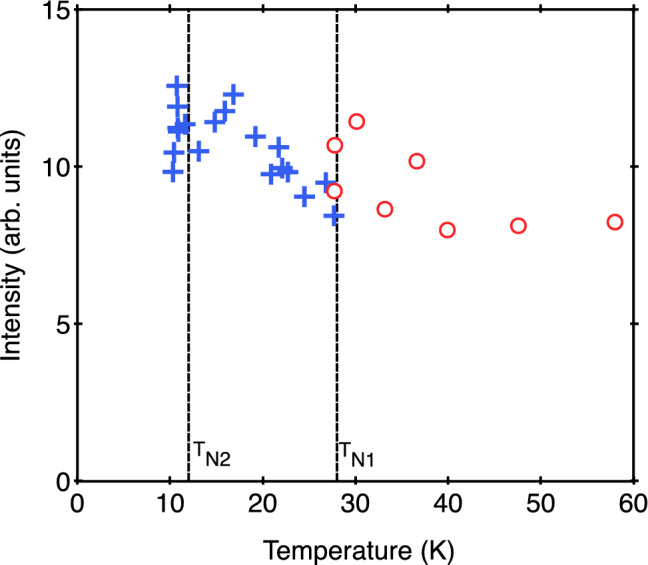
Temperature dependence of the E_1g_ mode intensity from Raman scattering measurements.

Some further properties obtained for the E_1g_ phonon are shown in Figs. 6 and 7, where the mode damping and intensity, respectively, are plotted versus temperature. The E_1g_ phonon damping shows no anomaly at *T*_N1_ and continues to decrease smoothly with decreasing temperature, which is a normal behavior for a phonon in a nonmagnetic crystal in any case, but unexpectedly it then sharply increases below the 3D magnetic ordering temperature T_N2_ (see Fig. 6). The E_1g_ phonon Raman intensity exhibits an even more complicated variation with temperature (see Fig. 7). These characteristics would warrant further attention.

## Conclusions

In conclusion, we have demonstrated unusual properties for the spin-phonon interactions in influencing the phonon frequencies for two modes in RbCoCl_3_ with frequencies comparable to those of the low-frequency magnons. Our theoretical analysis, with modifications included to take account of the large Ising-like character demonstrated by the exchange interactions between spins, enabled the spin-phonon coupling coefficients to be deduced. This newly-observed aspect of the crystal properties at, and below *T*_N1_, deserves further investigation with high resolution x-ray diffraction and both inelastic and elastic neutron scattering. Such magnetic-ordering-related lowering of the crystal structure has been noted previously for CsCoBr_3_^24^, but nothing transpires as drastically at *T*_N1_ as that occurring in RbCoCl_3_.

## Methods

The Raman measurements were performed on a single crystal of dark-blue colored RbCoCl_3_. The sample was mounted in a Thor S500 continuous-flow cryostat, where the crystal temperature could be controlled to within 0.1 K. The Raman scattering spectra were excited with 50 mW of 476.5 nm argon laser light, analyzed with a Spex 14018 double monochromator, and detected with a cooled RCA 31034A photomultiplier. The Raman signal was recorded at right angles to the incident light in a X(..)Y scattering geometry, where the Y axis was chosen to be normal to the crystal ($1\overline{1 }20$) cleavage plane and the Z axis was along the crystal *c* axis.

## Data Availability

All data generated or analysed during this study are included in this published article.
